# Analysis of HIV-1 Vpr determinants responsible for cell growth arrest in *Saccharomyces cerevisiae*

**DOI:** 10.1186/1742-4690-1-21

**Published:** 2004-08-16

**Authors:** Xiao-Jian Yao, Nicole Rougeau, Ghislaine Duisit, Julie Lemay, Éric A Cohen

**Affiliations:** 1 Laboratoire de Rétrovirologie Humaine, Département de Microbiologie et Immunologie, Faculté de Médecine, Université de Montréal, Montréal, Québec H3C 3J7, Canada; 2 Current address : Dept. of Medical Microbiology, University of Manitoba, Basic Medical Sciences Building, 730 William Avenue, Winnipeg, Manitoba R3E 0W3, Canada

## Abstract

**Background:**

The HIV-1 genome encodes a well-conserved accessory gene product, Vpr, that serves multiple functions in the retroviral life cycle, including the enhancement of viral replication in nondividing macrophages, the induction of G2 cell-cycle arrest, and the modulation of HIV-1-induced apoptosis. We previously reported the genetic selection of a panel of di-tryptophan (W)-containing peptides capable of interacting with HIV-1 Vpr and inhibiting its cytostatic activity in *Saccharomyces cerevisiae *(Yao, X.-J., J. Lemay, N. Rougeau, M. Clément, S. Kurtz, P. Belhumeur, and E. A. Cohen, J. Biol. Chem. v. 277, p. 48816–48826, 2002). In this study, we performed a mutagenic analysis of Vpr to identify sequence and/or structural determinants implicated in the interaction with di-W-containing peptides and assessed the effect of mutations on Vpr-induced cytostatic activity in *S. cerevisiae*.

**Results:**

Our data clearly shows that integrity of N-terminal α-helix I (17–33) and α-helix III (53–83) is crucial for Vpr interaction with di-W-containing peptides as well as for the protein-induced cytostatic effect in budding yeast. Interestingly, several Vpr mutants, mainly in the N- and C-terminal domains, which were previously reported to be defective for cell-cycle arrest or apoptosis in human cells, still displayed a cytostatic activity in *S. cerevisiae *and remained sensitive to the inhibitory effect of di-W-containing peptides.

**Conclusions:**

Vpr-induced growth arrest in budding yeast can be effectively inhibited by GST-fused di-W peptide through a specific interaction of di-W peptide with Vpr functional domain, which includes α-helix I (17–33) and α-helix III (53–83). Furthermore, the mechanism(s) underlying Vpr-induced cytostatic effect in budding yeast are likely to be distinct from those implicated in cell-cycle alteration and apoptosis in human cells.

## Background

Human immunodeficiency virus 1 (HIV-1) Vpr is a small virion-associated protein that is incorporated into virions through a specific interaction with the p6 domain of the p55^gag ^precursor protein [1,2]. Increasing evidence suggests that Vpr plays important roles during HIV-1 replication and pathogenesis. First, virion-associated Vpr has been shown to act early in viral infection as a facilitator of HIV-1 preintegration complex (PIC) entry through the limiting nuclear pore. This activity of Vpr is thought to be responsible for Vpr's ability to enhance HIV-1 replication in nondividing cells, most notably in terminally differentiated macrophages [3-5]. Second, expression of Vpr induces a G2 cell cycle arrest, which is thought to indirectly enhance viral replication by increasing transcription from the HIV-1 long terminal repeat (LTR) [6,7].

Even though the molecular mechanism of Vpr-mediated cell-cycle G2 arrest is still obscure, it has been known that Vpr expression leads to inactivation of the mitotic p34cdc2/cyclinB complex in human cells [8,9] as well as in fission yeast *Schizosaccharomyces pombe (Sc. Pombe) *[10-14]. Involvement of protein phosphatase 2A (PP2A), Wee1, Cdc25C, and 14-3-3 proteins has also been implicated [8-12,14] but the host cell proteins directly engaged by Vpr are not yet identified. Noteworthy, HIV-1 Vpr expression induces also a growth arrest in *Saccharomyces (S.) cerevisiae *[15-17]. Deletion mapping studies showed that the C-terminal 33 amino acids, including the H(S/F)RIG motif, contributed to this cytostatic effect [15,18]. Although this region has also been implicated in Vpr-mediated cell-cycle dysregulation in mammalian and *S. Pombe *cells [19-25], the molecular mechanism of Vpr-growth arrest in budding yeast is thought to be distinct since growth arrest occurs independently of any evident block at the G2/M transition [16]. Accordingly, it has been reported that the G2/mitosis transition in budding yeast is regulated differently than in mammalian cells and fission yeast [26,27]. Indeed, Vpr cytostatic effect observed in *S. cerevisiae *has been proposed to result from gross mitochondrial dysfunction [17] and/or cytoskeletal defects [16], rather than a cell cycle G2 arrest.

In addition to nuclear import and cytostatic activities, HIV-1 Vpr exhibits cytotoxic properties. Elevated intracellular expression or addition of extracellular Vpr or derived peptides results in proapoptotic effects in human cells including neurons [6,28,29] as well as cytotoxicity in budding and fission yeasts [30,31]. Jacotot *et al*. have provided evidence indicating that extracellular Vpr or peptides derived from Vpr C-terminus induce mitochondrial dysfunction in human cells by a mechanism involving a specific binding to the adenine nucleotide translocator (ANT), a component of the permeability transition pore complex (PTPC) in the mitochondrial membrane. The resulting mitochondrial membrane permeabilization (MMP) leads to a decreased membrane potential and the release of cytochrome c and apoptosis inducing factor (AIF) [32,33]. This Vpr-mediated MMP is thought to initiate cell death through both caspase-dependent and independent mechanisms in human cells as well as cytotoxicity in budding yeast [32-37]. In addition, it has also been shown that extracellular Vpr is capable of forming cation-selective ion channels in planar lipid bilayers, which can depolarize intact cultured neurons, thus leading to cell death [28].

In a previous report, we have shown that expression of genetically-selected glutathione-S-transferase (GST)-fused di-tryptophan (di-W)-containing peptides inhibited Vpr-mediated growth arrest in *S. cerevisiae *presumably by interacting with Vpr [38]. Interestingly, these, di-W-containing peptides were also able to inhibit Vpr biological activities, including nuclear import, cell cycle G2 arrest and apoptosis, in mammalian cells or HIV-1 infected T cells [38]. Even though the inhibitory effect of these di-W-containing peptides correlated with their ability to interact with Vpr in budding yeast, the detailed mechanism underlying their mode of action remains to be defined. In addition, it is still unclear whether the growth arrest activity of Vpr in budding yeast is related to specific biological activities of Vpr in human cells. In this study, we have performed a mutagenic analysis of Vpr to identify Vpr domains important for di-W peptide binding and cytostatic activity in *S cerevisiae*. Results reveal that the inhibitory di-W-containing peptides target specifically a functional domain of Vpr directly involved in growth arrest in budding yeast. Furthermore, several previously well-characterized Vpr mutants unable to induce cell-cycle dysregulation and/or apoptosis in mammalian cells still exhibit strong growth arrest activity in budding yeast, indeed suggesting that Vpr carries out distinct functions in *S. cerevisiae*.

## Results

### Analysis of Vpr sequence and/or structural determinants implicated in the interaction with di-W-containing peptides

**Figure 1:**
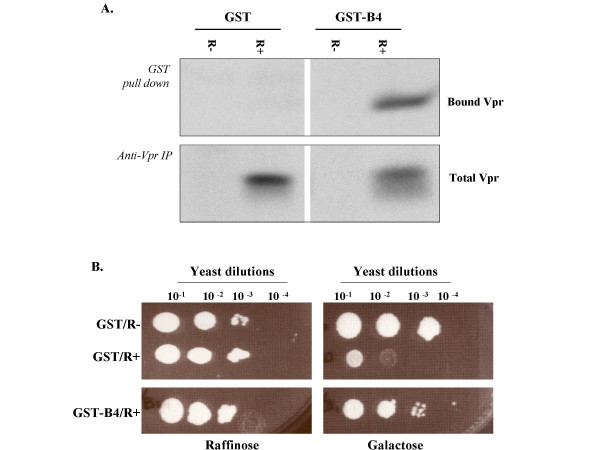
GST-B4 peptide binds to HIV-1 Vpr in *S. cerevisiae *and rescues cell growth. (A) GST pull-down from yeast extracts. *S. cerevisiae *HP16 strain co-transformed with GST or GST-B4 plasmids and (R+) or (R-) Vpr expressor were metabolically-labeled with 150 μCi of ^35^S-Translabel in Vpr-inducible medium. Half volume of the cell extract was used for GST pull-down, while the remaining lysates were subjected to immunoprecipitation with polyclonal anti-Vpr antiserum. Total and GST-bound radiolabeled Vpr proteins were detected by autoradiography after SDS-PAGE. (B) GST-B4 suppression of Vpr-induced cell growth arrest. Yeast co-transformants were grown in non-inducible selective medium for two days. Similar number of yeast cells were then serially diluted, spotted onto either Vpr non-inducible (Trp^-^/Ura^-^, 2% raf) or Vpr-inducible plates (Trp^-^/Ura^-^, 2% gal) and incubated for 3 to 5 days to evaluate their growth rates. This data is representative of results obtained in two independent experiments

We have previously used a genetic selection system in *S. cerevisiae *budding yeast and selected a panel of di-W-containing GST-peptides that specifically inhibit Vpr-mediated yeast growth arrest function presumably through their ability to bind HIV-1 Vpr [38]. In this study, we further investigated the molecular mechanism of this inhibition using a newly selected GST-fused di-W peptide WWSFKSV (GST-B4), which displayed an enhanced ability to bind Vpr and inhibit its growth arrest activity in budding yeast (Fig. 1A and 1B).

**Figure 2:**
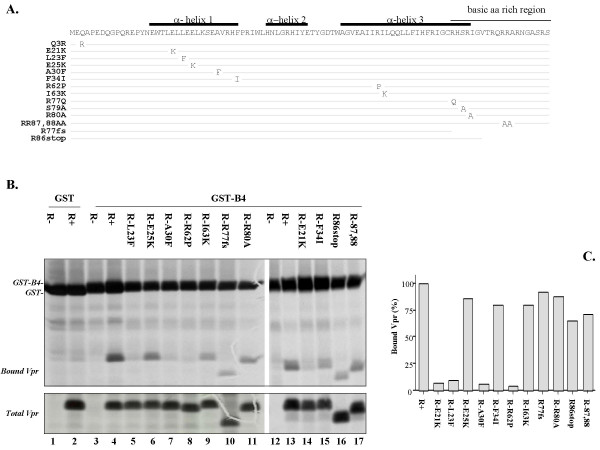
HIV-1 Vpr mutants exhibit differential GST-B4 binding abilities. Each Vpr mutant used in this study with the exact location of the introduced mutation is described (A). (B) GST pull-down using a panel of Vpr mutants. Assays were performed as described in Fig. 1A. Protein extracts were prepared from radiolabeled cells expressing GST (lanes 1–2) or GST-B4 proteins (lanes 3–17) alone (R-), or in presence of wild-type Vpr (R+) or different mutant proteins, as indicated. Vpr bound to GST-B4 (upper panel) and the total amount of Vpr as determined using immunoprecipitation with anti-Vpr antiserum (lower panel) were separated by SDS-PAGE and detected after autoradiography. (C) The percentage of GST-B4-bound Vpr relative to the total amounts of Vpr for each mutant was quantified by autoradiography scanning and the level of wild type Vpr bound to GST-B4 was arbitrarily set as 100%. These data are representative of at least two independent experiments.

Structural studies performed with synthetic forms of Vpr indicate that Vpr is characterized by a well-defined gamma turn (14–16)-alpha helix (α-helix I: 17–33)-turn (34–36), followed by an alpha helix(α-helix II: 40–48)-loop (49–54)-alpha helix (α-helix III: 55–83) domain and ends with a very flexible C-terminal arginine-rich sequence [39]. The α-helical determinants where shown to be required for Vpr virion incorporation, nuclear localization and oligomerization [39-44] and are believed to be involved in heterologous protein binding [45]. The arginine rich C-terminal region of Vpr has not been shown to have a predicted structure, however this region harbors protein phosphorylation sites and plays an important role in cell cycle G2 arrest and the nuclear localization of the protein in mammalian cells [6,31,46]. To further investigate the sequence and/or structural requirement of Vpr for GST-B4 binding, mutations were introduced in p424Gal1-Vpr expressor to target different regions of Vpr (Fig. 2A). The N-terminal Q3R mutant was shown to affect Vpr proapoptotic activity during HIV-1 replication [47]. Four amino acids Glu21, Leu23, Glu25 and Ala30 were separately changed to Lys or Phe (E21K, L23F, E25K and A30F) in order to disrupt the amphipaticity of the first α-helix [39] (Fig. 2A). The F34I was introduced in a γ-turn region which is just after the α-helix I [39]. The R62P and I63K mutations introduced in the third helix were aimed at interfering with the integrity of the α-helix and are known to abolish Vpr nuclear localization [41]. Four mutants in the C-terminal region, including, R77Q, S79A, R80A and R87, 88, were generated to replace positively-charged arginine residues or to remove the critical phosphorylation site (Ser 79) of the protein. Vpr mutants S79A and R80A were reported to be defective for cell cycle G2 arrest activity in mammalian cells, while the proapoptotic activity of R77Q was severely attenuated [6,24,48]. In addition, a frameshift mutation (R77fs) [40] and a truncation mutation (R86stop), which prematurely terminate the protein at amino acid 77 and 86 respectively were also constructed.

To determine the impact of the Vpr mutations on GST-B4 peptide binding, HP-16 yeast co-transformed with mutated-Vpr expressors and either GST or GST-B4 vectors were radio-labeled in Vpr-inducible medium and subjected to GST pull-down assays (Fig. 2B), as described in Materials and Methods. Moreover, the amount of wild type Vpr or each mutant bound to GST-B4 peptide was evaluated by laser densitometric scanning of bands in autoradiograms and normalized to the total amounts of Vpr and GST proteins that were expressed in each transformants. The amounts of wild type Vpr bound to GST-B4 was arbitrarily set as 100% (Fig. 2C). Results of figure 2B reveal that all Vpr mutants were expressed at comparable levels, as determined by Vpr immunoprecipitation of induced-cell lysates with the exception of Vpr (R77fs), which indeed was previously reported to be less stable than wild type Vpr [40] (Fig. 2B, lower panel). While no Vpr interacted with GST (Fig. 2B, upper panel, lane 2), similar levels of wild type Vpr, E25K, F34I, I63K, R77fs, R80A, R87, 88 and R86stop mutants were pulled-down with GST-B4 (Fig. 2B, upper panel and 2C). Similar results were obtained for Vpr mutants Q3R, R77Q, S79A (data not shown). In contrast, E21K, L23F, A30F and R62P mutants, which are respectively located in α-helix I and α-helix III regions, were not co-pulled down with GST-B4 (Fig. 2B (upper panel, lanes 5, 7, 8 and 14) and 2C). Taken together, these results suggest that the integrity of the N-terminal α-helix I and the α-helix III of Vpr are crucial for GST-B4 binding, whereas the C-terminal domain is dispensable for the interaction.

### Vpr mutants defective for GST-B4 binding are unable to arrest yeast cell growth

**Figure 3:**
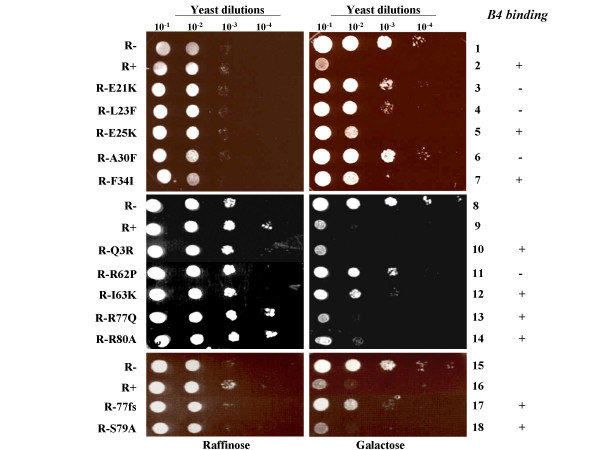
The growth arrest activity of different Vpr mutants. *S. cerevisiae *HP16 yeast was transformed with the p424Gal1 expressor alone (R-) or coding for wild-type (R+) or each mutant, as indicated at the left of photograph, and first grown in non-inducible medium for 2 days. Then, similar amounts of transformed yeast were serially 10× diluted and spotted onto either non-inducible (Trp^-^, 2% raf) or Vpr-inducible (Trp^-^, 2% gal) plates and incubated for 3 days to evaluate their growth rates. This data is representative of at least two independent experiments. The ability of each mutant to bind the B4 peptide is indicated on the right. (+) indicates efficient binding while (-) indicates absence of binding.

We next tested the growth arrest activity of these Vpr mutants in HP-16 yeast. Growing yeast cells transformed with the empty vector (R-), wild-type (R+) or mutated Vpr expressors were serially diluted and spotted onto either a Vpr non-inducible plate (Trp^-^, 2% raf) or a Vpr-inducible plate (Trp^-^, 2% gal). Cell growth was evaluated following an incubation of 3–5 days at 30°C (Fig. 3). In Vpr non-inducible plate, all yeast transformants grew at similar rate (Fig. 3, left panel). Upon galactose induction, while the empty vector (R-)-transformed yeast grew efficiently (Fig. 3, lanes 1, 8 and 15), the wild-type Vpr (lanes 2, 9 and 16), the Q3R mutant and all proteins mutated in the C-terminal region, including R77Q, S79A, R80A exhibited a profound growth arrest activity (Fig. 3, right panel (lanes 10,13, 14 and 18). Similar results were obtained for R-87,88 and R86stop mutants (data not shown), indicating that the C-terminal arginine-rich region of Vpr is not involved in budding yeast growth arrest activity. Of note, R77fs showed an impaired growth arrest activity (Fig. 3, lane 17), which is most likely due to the shorter half-life of this truncated protein, as reported before [40]. In contrast, expression of helices I and III Vpr mutants, E21K, L23F, A30F and R62P, which displayed a strong attenuation of binding to GST-B4, did not lead to HP-16 budding yeast growth arrest (Fig. 3, right panel, lanes 3, 4, 6 and 11). On the contrary, helix I or III mutants E25K, F34I, and I63K, which were able to interact with GST-B4, still exhibited growth arrest activity, even though at reduced levels as compared to wild-type Vpr (Fig. 3, right panel, 5, 7 and 12). These results indicate that Vpr helices I and III represent an important functional domain involved in growth arrest in budding yeast.

### GST-B4 inhibits the cytostatic activity of Vpr mutants and rescues cell growth

**Figure 4:**
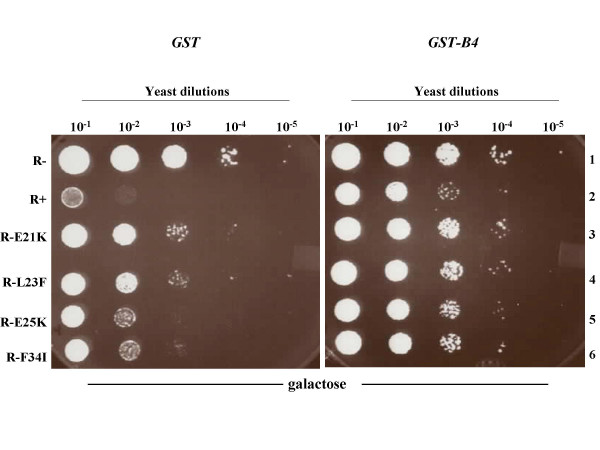
Comparison of GST-B4-mediated inhibition of the growth arrest activity of different Vpr mutants. *S. cerevisiae *HP16 yeast co-expressing GST (left panel) or GST-B4 (right panel) and a panel of representative Vpr mutants, as indicated, were serially 10× diluted and plated on Vpr-inducible and selective (Trp^-^/Ura^-^, 2% gal) plates as described in Fig. 3. The respective cell growth was evaluated after a 3-day incubation. This data is representative for two independent experiments.

To further investigate the correlation between the inhibitory effect of GST-B4 and its Vpr-binding ability, GST or GST-B4 were co-expressed with two GST-B4-binding defective Vpr mutants E21K and L23F or with two GST-B4-binding competent mutants E25K and F34I in HP-16 yeast and the resulting cell growth was monitored in Vpr-inducible plates as described above. In agreement with the data of figure 3, in the presence of GST alone, mutants E25K and F34I induced significant yeast growth arrest, while such activity was severely impaired for B4-binding defective mutants E21K and L23F (Fig. 4, left panel). In contrast, GST-B4 co-expression strongly inhibited the growth arrest activity of the wild type Vpr, E25K and F34I mutants and indeed restored their cell growth at a level comparable to that of yeast cells expressing E21K or L23F (Fig. 4, right panel). A weak inhibitory activity of B4 was also observed with mutants E21K and L23F (lanes 3 and 4). It is possible that this may reflect a weak or instable interaction between B4 and Vpr mutants E21K and L23F, which could not be clearly detected in the binding experiments (Fig. 3). Overall, these results clearly indicate that GST-B4 specifically binds to structural determinants that are important for inducing cell growth arrest. Moreover, as described previously (38), the binding efficiency of B4 peptides correlates with the extent of the peptide inhibitory activity.

## Discussion

We have previously shown that GST-fused di-W-containing peptides were able to interact with HIV-1 Vpr and as a result inhibit its multiple functions in budding yeast as well as in HIV-1 infected T cells [38]. In the present study we have further investigated the sequence and/or structural determinants required for Vpr/peptide interaction and determined their impact on Vpr cytostatic activity in budding yeast. Results clearly show that GST-fused B4 peptide interaction with Vpr involves the α-helical I and III structure of Vpr. Mutations affecting the integrity of these helical regions not only interfered with the interaction with GST-B4 peptide, but also failed to induce a cytostatic activity in budding yeast. Furthermore, Vpr mutants, including Q3R, R77Q, R80A and S79A, yet defective for cell-cycle arrest or apoptosis in mammalian cells, still induced a growth arrest in *S. cerevisiae *and displayed sensitivity to GST-B4 inhibition. Overall, these results indicate that GST-fused di-W-containing peptides directly target functional domains of HIV-1 Vpr responsible for inducing growth arrest in budding yeast and strongly suggest that the mechanism(s) underlying Vpr-induced cytostatic effect in budding yeast are distinct from those implicated in cell-cycle alteration and apoptosis in mammalian cells.

Previous reports have indicated that the Vpr cytostatic activity in *S. cerevisiae *budding yeast was attributed to its last 63–96 amino acid (aa) and the critical domain was located in a conserved C-terminal HFRIGCRHSRIG sequence from aa 71 to 82 [15]. In contrast, our results showed that expression of a truncated Vpr encompassing aa 1 to 77 was sufficient to induce growth arrest (Fig. 3), suggesting that the sequence of HFRIG (aa 71 to 75), but not HSRIG (aa 78 to 82) and other C-terminal region of Vpr, may constitute one important determinant for this Vpr-induced phenotype. Consistently, a mutagenic analysis by Berglez *et al*., revealed that substitution mutations of aa His71 or Gly75 in this HFRIG sequence abolished Vpr cytostatic effect in budding yeast [18]. Interestingly, our analysis clearly reveal that the N-terminal α-helix I and the α-helix III are both contributing to Vpr cytostatic effect, which is in agreement with a previous finding by Gu *et al *showing that the Vpr F34I mutant was unable to induce a growth arrest phenotype in budding yeast [16]. On the basis of the Vpr NMR structure reported by Wecker *et al *[39], mutations E21K, L23F, E25K and A30F located within the α-helix I (from aa 17 to 33) were designed to disrupt either the negatively-charged cluster or the hydrophobic interface. With the exception of E25K mutant, all other mutations in this N-terminal α-helical region lead to the loss of Vpr cytostatic function (Fig. 3). In addition, disruption of the third α-helix by introduction of a proline at position 62 (R62P) suppressed Vpr-induced growth arrest, suggesting that integrity of α-helices I and III was required for Vpr cytostatic activity in budding yeast. It was also noted that E25K and I63K still induced a low level of growth arrest compared to other helical region mutants (Fig. 3). It could be possible that E25K is somewhat external to the spatially-aligned acidic cluster D17-E21-E24 [39], and may be therefore less critical. Similarly, the I63K mutation may have a minor impact on the tridimensional structure of helix III as compared to the introduction of a proline residue as with the R62P Vpr mutant.

One striking observation of this study is that the four mutants (E21K, L23F, A30F and R62P) located in the α-helical I and III regions of Vpr, which were defective for the cytostatic activity in budding yeast (Fig. 3) also lost the ability to interact with the inhibitory GST-B4 peptide (Fig. 2). It indicates that GST-B4 directly targets a critical functional domain, possibly a structural cluster comprising both of α-helical I and III, that is responsible for cytostatic activity. Interestingly, the sequence of GST-B4 (GST-WWSKKSV) reveals that, in addition to a conserved di-W motif [38], it also harbors an overlapping WxxF motif, which has been previously isolated by phage-display as a Vpr-binding motif and is present in Vpr-interacting protein uracil DNA glycosylase (UDG) [49]. Coincidentally, a bipartite domain encompassing Vpr amino acids 15–27 and 63–77 was also shown to be involved in UDG binding [50]. Based on these observations, it appears that similar regions of Vpr are involved in binding to UDG and GST-B4 through targeting of a WxxF element. However, E25K and F34I mutants, which were shown to be defective for UDG binding in two-hybrid assays [21], were still able to interact with GST-B4 *in vivo*. Such a difference may specifically rely on the hydrophobic di-W motif, which is not present in UDG [49].

Up to date, how HIV-1 Vpr induces a growth arrest in budding yeast remains an open question. During HIV-1 replication, the expression of Vpr has been shown to induce a cell cycle G2 arrest resulting from the inactivation of the mitotic p34cdc2/cyclinB complex [51]. In contrast, Vpr-mediated growth arrest in budding yeast is thought to occur through a distinct mechanism(s), since it occurs independently of any evident block at the G2/M transition [16]. In this study, we tested a panel of well-characterized Vpr mutants for their ability to growth arrest HP-16 budding yeast. Interestingly, Vpr mutants (S79A and R80A) which were previously shown to be as stable as wild type Vpr but defective for cell-cycle G2 arrest in human cells [6,19,24] still induced strong growth arrest in budding yeast. Conversely, L23F, and R62P mutants, which are competent for cytostatic effect in mammalian cells, [20,41] were unable to block yeast growth. Therefore, it can be concluded that Vpr structural determinants required for growth arrest in *S. cerevisiae *and human cells are clearly distinct, implying that different molecular mechanisms governs Vpr activities in these different cell species. Moreover, our study also demonstrates that Vpr cytostatic effect in budding yeast is not related to the cytotoxic activity of the viral protein. Vpr exhibits different cytotoxic properties that implicate distinct domains of the viral protein. First, wild-type Vpr and its first 40 N-terminal amino acids can form cation-selective ion channels in lipid bilayers [28,52]. Depolarization of the plasma membrane resulting from inward sodium current eventually induces killing of polarized cells such as neurons. On the other hand, apoptosis in T cells is thought to be triggered by transduction of full-length Vpr or its C-terminal 52–96 moiety into cells and involves mitochondrial membrane permeabilization [33,53,54]. Resulting loss of mitochondrial transmembrane potential then induces the release of apoptogenic proteins, leading to caspase-dependent (37,55,48) or caspase-independent [55] cell killing. The fact that both 17–33 and 55–83 alpha-helices are required for growth arrest in *S. cerevisiae *strongly suggests that the cytostatic effect observed in budding yeast is mechanistically distinct from effects resulting from ion channels formation or mitochondria permeabilization. Consistently, Q3R, R80A, R77Q Vpr mutants, which were previously shown to be as stable as wild type Vpr, but yet defective for apoptosis induction in human cells [6,47,48] were still able to block yeast growth in a B4-sensitive way.

## Conclusions

Taken together, the results presented here provide evidence that Vpr triggers growth arrest in budding yeast by an undefined mechanism that is unrelated to Vpr-induced G2 arrest and apoptosis in mammalian cells. This Vpr-induced budding yeast growth arrest can be effectively inhibited by GST-fused di-W peptide through an interaction of di-W peptide with Vpr functional domain, which includes α helix I and III. These observations would support a model in which, Vpr interacts with a di-W-containing protein in *S. cerevisiae *to induce yeast growth arrest. The question that still remains unanswered at this point is whether this Vpr cytostatic activity in budding yeast can also play an important role during HIV-1 replication and viral pathogenesis and further investigations are currently underway to address this question.

## Materials and methods

### Yeast strain

The *S. cerevisiae *yeast strain used in this study was the protease-deficient HP-16 strain (*MAT∝ ura3-52 his3Δ1 leu2 trp1Δ63 prb1-1122 pep4-3 prc1-407*) [56]. Plasmid transformation was performed using the lithium acetate method [57].

### Plasmids, antisera and chemicals

The HIV-1 Vpr yeast expression plasmid (p424Gal1-Vpr) and the negative control plasmid p424Gal1-R^- ^have been previously described [38]. To generate different p424Gal1-Vpr mutant expression plasmids, each of Vpr mutant cDNAs (Fig. 2A) was generated by a two-steps polymerase chain reaction (PCR)-based method [40] by using a 5'-primer (5'-CTGCTAGCGGATAGATGGGA-3') harboring a *BamH*I site in front of the Vpr initiation codon, a 3'-primer (5'-GCATCGCTCGAGGATCTACTGGC-3') containing a *Xho*I site after the stop codon of Vpr and the complementary oligonucleotide primers containing the desired mutations. Amplified Vpr cDNA harboring specific mutations were then cloned into the p424Gal1 vector at *BamH*I/*Xho*I sites. The Vpr mutants L23F, E25K, A30F, R62P, I63K, R77Q, R77fs and R80A were previously described [6,41,48]. The pPGK-GST plasmid was described previously [38] while the pPGK-GST-B4 expressor was isolated and purified from an *S. cerevisiae *HP-16 yeast colony that was resistant to HIV-1 Vpr-mediated growth arrest as previously described [38].

The rabbit anti-Vpr polyclonal serum was raised against bacterially expressed recombinant Vpr as described previously [58]. Galactose, raffinose and glucose were purchased from Sigma Inc.

### Evaluation of the growth arrest activity of Vpr mutants and the anti-Vpr activity of GST-peptide in budding yeast

The experimental procedures to evaluate protein expression, Vpr growth arrest activity and the anti-Vpr activity of GST-fused di-W peptide were described previously [38]. Briefly, HP-16 yeast cells transformed with p424Gal1-wild-type/mutant Vpr plasmids or co-transformed with Vpr expressors and pPGK-GST-B4 plasmid were first grown in a Vpr non-inducible selective medium (Trp^- ^or Trp^-^/Ura^-^, 2% raffinose (raf^+^)) for 2 days. Then, suspensions of transformed HP-16 yeast cells (adjusted at similar cell densities) were serially diluted and spotted onto either a Vpr non-inducible plate (Trp^- ^or Trp^-^/Ura^-^, 2% raf) or a Vpr-inducible plate (Trp^- ^or Trp^-^/Ura^-^, 2% gal) to evaluate the growth of each co-transformed HP16 population.

### GST pull-down assay and anti-Vpr immunoprecipitation

HP16 co-transformants were radiolabeled with 150 μCi of ^35^S-Translabel (ICN Inc.) in Vpr-inducible medium and lysed in CHAPS buffer as previously described [38]. Cell extracts were then subjected to GST pull-down assay [4,38]. Briefly, lysates were incubated with glutathione-sepharose 4B beads (Amerham Pharmacia Biotech Inc) for 2 hours at 4°C. Beads were washed 3 times and the radiolabeled protein complexes were eluted with an elution buffer (100mM reduced gluthathione, 120 mM NaCl, 100 mM Tris-HCl pH 8.5) by gentle shaking at 4°C for 1 hour. Eluted protein complexes were separated by SDS-PAGE and detected by autoradiography. For Vpr expression analysis, aliquots of labeled yeast lysates were immunoprecipitated with anti-Vpr antibodies as described previously [38,40].

## Authors' contributions

X-J Y designed the experiments, constructed most Vpr mutants and wrote the manuscript. NR carried out the binding assays and tested the effect of Vpr mutants on yeast cells growth. JL selected and characterized the B4 GST-di-W-containing peptide. GD participated in the design of the study and critically evaluated the manuscript. EAC participated in the design of the study and coordinated it. All authors read and approved the final manuscript.
